# Sonochemotherapy: from bench to bedside

**DOI:** 10.3389/fphar.2015.00138

**Published:** 2015-07-10

**Authors:** Bart H. A. Lammertink, Clemens Bos, Roel Deckers, Gert Storm, Chrit T. W. Moonen, Jean-Michel Escoffre

**Affiliations:** ^1^Image Guided Therapy, Imaging Division, University Medical Center UtrechtUtrecht, Netherlands; ^2^Department of Pharmaceutical Sciences, Faculty of Science, Utrecht UniversityUtrecht, Netherlands; ^3^Targeted Therapeutics, MIRA Institute for Biomedical Technology and Technical Medicine, University of TwenteEnschede, Netherlands

**Keywords:** ultrasound, microbubble, sonoporation, chemotherapeutic drug, drug delivery, sonochemotherapy

## Abstract

The combination of microbubbles and ultrasound has emerged as a promising method for local drug delivery. Microbubbles can be locally activated by a targeted ultrasound beam, which can result in several bio-effects. For drug delivery, microbubble-assisted ultrasound is used to increase vascular- and plasma membrane permeability for facilitating drug extravasation and the cellular uptake of drugs in the treated region, respectively. In the case of drug-loaded microbubbles, these two mechanisms can be combined with local release of the drug following destruction of the microbubble. The use of microbubble-assisted ultrasound to deliver chemotherapeutic agents is also referred to as sonochemotherapy. In this review, the basic principles of sonochemotherapy are discussed, including aspects such as the type of (drug-loaded) microbubbles used, the routes of administration used *in vivo*, ultrasound devices and parameters, treatment schedules and safety issues. Finally, the clinical translation of sonochemotherapy is discussed, including the first clinical study using sonochemotherapy.

## Introduction

Cancer presents the second leading cause of death in the European Union with 3.45 million new cases of cancer and 1.75 million deaths from cancer in 2012 (Ferlay et al., 2013). Although a lot of progress has been made in the treatment of several cancers, many types of cancer are still lacking effective treatment options. Surgery, radiotherapy, and chemotherapy are the standard treatment possibilities and they are often combined to improve patient outcome.

While for most advanced cancers, chemotherapy remains the treatment of choice, it is rarely curative for solid tumors (Qin et al., 2015). To be successful, sufficient quantities of chemotherapeutic drugs have to reach the interior of tumor cells. Most small molecular weight chemotherapeutics (<4 kDa) are rapidly cleared from the circulation (e.g., t_1/2_ < 15 min for 5-fluorouracil, 5-FU), which is a limiting factor for drug accumulation in the tumor. In addition to challenges related to the physicochemical properties of drugs, tumors also possess physiological barriers (Jain, 2001). Contrary to healthy tissues, tumor tissues have a high interstitial fluid pressure (IFP), which is related to the lack of functional lymphatics and the leaky tumor vasculature (Boucher et al., 1990). These high pressures establish an outward fluid motion from the core of the solid tumor to the periphery and reduce fluid infiltration across the vascular wall. Thus, even if the leaky vasculature permits drug extravasation, diffusion-driven drug penetration deeper into the tumor tissue is severely restricted due to the high IFP. The increase in mean distance between vessels and tumor cells following tumor growth is another constraint for sufficient delivery of drugs. High tumor cell proliferation results in tumor cells forcing vessels apart, leading to a decrease in vascular density and a limitation in the access of drugs to distant tumor cells (Minchinton and Tannock, 2006). In addition, the presence of high levels of extracellular matrix limits the interstitial transport of drugs (Weinberg, 2014). Altogether these barriers oppose sufficient and uniform distribution of drugs in solid tumors, thereby limiting the therapeutic success of chemotherapy.

In addition, reaching the target site is not a guarantee that a drug will be effective. As most chemotherapeutic drugs need to enter the cell to become active, they need to pass the cell membrane. For several hydrophilic and charged drugs, e.g., bleomycin, this is a serious challenge and requires active uptake through plasma membrane transporters, which are not always present in the target cells (Pron et al., 1999).

In order to improve the efficiency of anti-cancer chemotherapeutics, physical methods including electroporation, laser, and magnetic fields have been developed (Sersa et al., 2008; Podaru et al., 2014; Sklar et al., 2014). The general principle of physical methods is based on the transient disruption of endothelial barrier and tumor cell membrane in order to facilitate the drug extravasation and the drug uptake into the endothelial and tumor cells. In recent years, research in the field of microbubble-assisted ultrasound (also known as sonoporation) aimed at delivering therapeutic molecules *in vitro* and *in vivo* has grown rapidly (Aryal et al., 2014; Azagury et al., 2014; Kiessling et al., 2014; Rychak and Klibanov, 2014; Unga and Hashida, 2014; Unger et al., 2014). Microbubble-assisted ultrasound transiently increases the permeability of biological barriers, such as blood vessel walls (i.e., drug extravasation) and cellular membranes (i.e., cellular uptake of drugs), thus enhancing the local delivery of therapeutic molecules across these barriers in the targeted region (Lentacker et al., 2014). Nowadays, the great potential of this modality for cancer therapy is clearly shown in an increasing number of publications on *in vitro* and *in vivo* drug delivery using microbubble-assisted ultrasound (**Tables 1** and **2** respectively). This method is a non-invasive, easy to apply, and cost-effective treatment modality, that can be used to deliver a wide range of anticancer molecules including low molecular weight chemotherapeutic agents (sonochemotherapy), nucleic acids and monoclonal antibodies to a target site, e.g., tumor (Escoffre et al., 2013c; Ibsen et al., 2013; Unga and Hashida, 2014). In addition, this method offers the possibility to treat superficial (e.g., skin) as well as deep organs (e.g., brain, liver, prostate), under the guidance of medical imaging modalities (magnetic resonance imaging, ultrasound imaging; Kinoshita et al., 2006; Deckers and Moonen, 2010; Lammers et al., 2015).

**Table 1:** *In vitro* sonochemotherapy.

Reference	Cell line	Drug (free vs. MB-loaded)	Microbubble	Ultrasound (US) parameters				Outcome vs. drug alone
				Frequency	Intensity	Duty cycle	Time	
Iwanaga et al. (2007)	Ca9-22	Free bleomycin	Optison	1 MHz	1.0 W/cm^2^	10%	20 s	2.5-fold increase in apoptosis
Heath et al. (2012)	SCC-1, SCC-5, Cal27	Free cisplatin	Definity	1 MHz	0.5 MI	20%	5 min	≈50% increase in apoptosis
Escoffre et al. (2011)	U87MG, MDA-231	Free doxorubicin (DOX)	Vevo, BR14, SonoVue	1 MHz	400–800 kPa	40%	30 s	30–40% decrease in viability, depending on cell line
Sorace et al. (2012)	2LMP	Free paclitaxel (PTX)	Definity	1 MHz	1.0 MPa PNP	20%	5 min	50% increase in cell death
Hu et al. (2012)	BEL-7402	Free 10-HCPT (free)	Polymer	3.5 MHz	22.57 mW/cm^2^	ND	10 min	20–30% decrease in viability
Ren et al. (2013)	DLD-1	Docetaxel-loaded MB	Lipid	800 kHz	2.56 W/cm^2^	50%	10 min	40% increase in inhibition rate
Tinkov et al. (2010)	295/KDR	DOX-loaded MB	Lipid	1 MHz	1 W/cm^2^	50%	20 s	40% decrease in cell viability
Yan et al. (2013)	4T1	PTX-liposome loaded MB	Lipid	1 MHz	1.0 MPa	50%	1 min	20–30% decrease in viability
Deng et al. (2014)	MCF7/ADR	DOX-liposome loaded MB	Lipid	1 MHz	1.65 W/cm^2^	20%	15 s	Increased cellular accumulation and retention, 30% decrease in viability

*10-HCPT, 10-hydroxycamptothecin; ND, non-defined; PNP, peak-negative-pressure*.

**Table 2:** *In vivo* sonochemotherapy.

Reference	Tumor (site, animal)	Drug	Microbubble	Administration route	US parameters				Outcome vs drug alone
					Frequency	Intensity	Duty cycle	Time	
Yan et al. (2013)	4T1 (s.c., mouse)	PTX-liposome loaded MB	Lipid	intravenous (i.v.)	2.25 MHz	1.9 MPa	1%	10 min	Fourfold increase it PTX accumulation, 2.5-fold decrease in tumor volume compared to PTX-loaded MB alone
Burke et al. (2014)	C6 (s.c., rat)	5FU-NPs loaded MB	Albumin	i.v.	1 MHz	1.2 MPa (PNP)	ND	Every 5 s for 60 min	Twofold decrease in tumor volume, increase in median survival (34 days vs. 26 days) compared to free 5FU
Fan et al. (2013)	C6 (i.c., rat)	VEGFR2-BCNU- loaded MB	Lipid	i.v. (infusion)	1 MHz	0.7 MPa	5%	1 min / sonication site	1.86-fold increase in it BCNU accumulation, threefold decrease in liver BCNU accumulation, 1.75-fold decrease in tumor volume, increase in median survival (>75 days vs. <40 days) compared to untargeted BCNU-loaded MB
Iwanaga et al. (2007)	Caco-9 (s.c., mouse)	Free Bleomycin	Optison	Intratumoral (i.t.; co-injection)	1 MHz	2 W/cm^2^	50%	2 min	Twofold decrease in tumor volume compared to free BLM
Kang et al. (2010)	VX2 (liver, rabbit)	Docetaxel-loaded MB	Lipid	i.v. (infusion)	0.3 MHz	2 W/cm^2^	50%	6 min	Threefold increase tumor inhibition, twofold increase in apoptosis, twofold decrease in proliferation compared to free Docetaxel
Li et al. (2012)	H22 (s.c., mouse)	10-HCPT loaded MB	Lipid	i.v.	1 MHz	2 W/cm^2^	50%	6 min	Sixfold increase in it 10-HCPT accumulation, twofold decrease in tumor volume compared to free 10-HCPT
Pu et al. (2014)	A2780/DDP (i.p. mouse)	LHRHa-PTX loaded MB	Lipid	intraperitoneal (i.p.)	0.3 MHz	1 W/cm^2^	50%	3 min	Twofold decrease in apoptotic index, twofold decrease in vessel number, twofold decrease in VEGF expression, 1.7-fold increase in caspase-3 expression, increase in survival median (>50 days vs. <40 days) compared to free PTX
Sonoda et al. (2007)	B16 (s.c., mouse)	Free BLM	Optison	i.t. (co-injection)	1 MHz	2 W/cm^2^	50%	4 min	Tumor eradication compared to free BLM
Treat et al. (2012)	9L (i.c., rat)	Free Doxil	Definity	i.v.	1.7 MHz	1.2 MPa	1%	1–2 min	1.5-fold decrease in tumor volume and median survival compared to free Doxil
Escoffre et al. (2013b)	U-87 MG (s.c., mouse)	Free Irinotecan	MM1	i.v.	1 MHz	0.4 MPa (PNP)	40%	3 min	Threefold decrease in tumor volume, twofold decrease in tumor perfusion, threefold increase necrosis, 35% decrease in mitosis index, no acute liver toxicity compared to free irinotecan
Ting et al. (2012)	C6 (i.c., rat)	BCNU-loaded MB	Lipid	i.v.	1 MHz	0.5–0.7 MPa	5%	1 min / sonication site	Fivefold increase in circulatory half-life of BCNU, fivefold decrease in liver accumulation, 13-fold decrease in tumor volume, 12% increase in median survival compared to free BCNU
Tinkov et al. (2010)	DSL6A (s.c., rat)	DOX-loaded MB	Lipid	i.v. (perfusion)	1.3 MHz	1.2 MPa	ND	Four ultrasound frames every four cardiac cycles	10-fold i.t. DOX accumulation, twofold decrease in tumor volume compared to DOX-loaded MB treatment alone

*s.c., subcutaneous; i.c., intracerebral; 10-HCPT, 10-hydroxycamptothecin; BLM, bleomycin; DOX, doxorubicin; ND, non-defined; PNP, peak-negative-pressure*.

This review first focuses on the biological effects of microbubble-assisted ultrasound (i.e., increasing plasma membrane- and vascular endothelium permeability) and subsequently on *in vitro* and *in vivo* chemotherapeutic drug delivery studies using microbubble-assisted ultrasound for cancer treatment. The limitations and future developments of sonochemotherapy will be further discussed.

## Microbubble-Assisted Ultrasound

**FIGURE 1:**
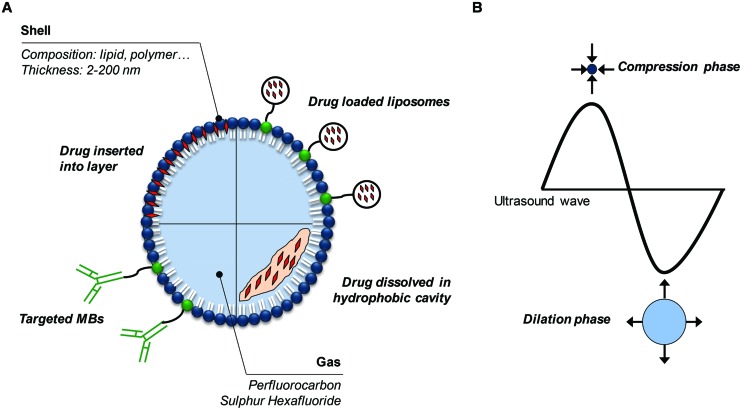
**Microbubbles and ultrasound. (A)** Different options for drug-loading or targeting of microbubbles. **(B)** Microbubble oscillations under ultrasound exposure.

**FIGURE 2:**
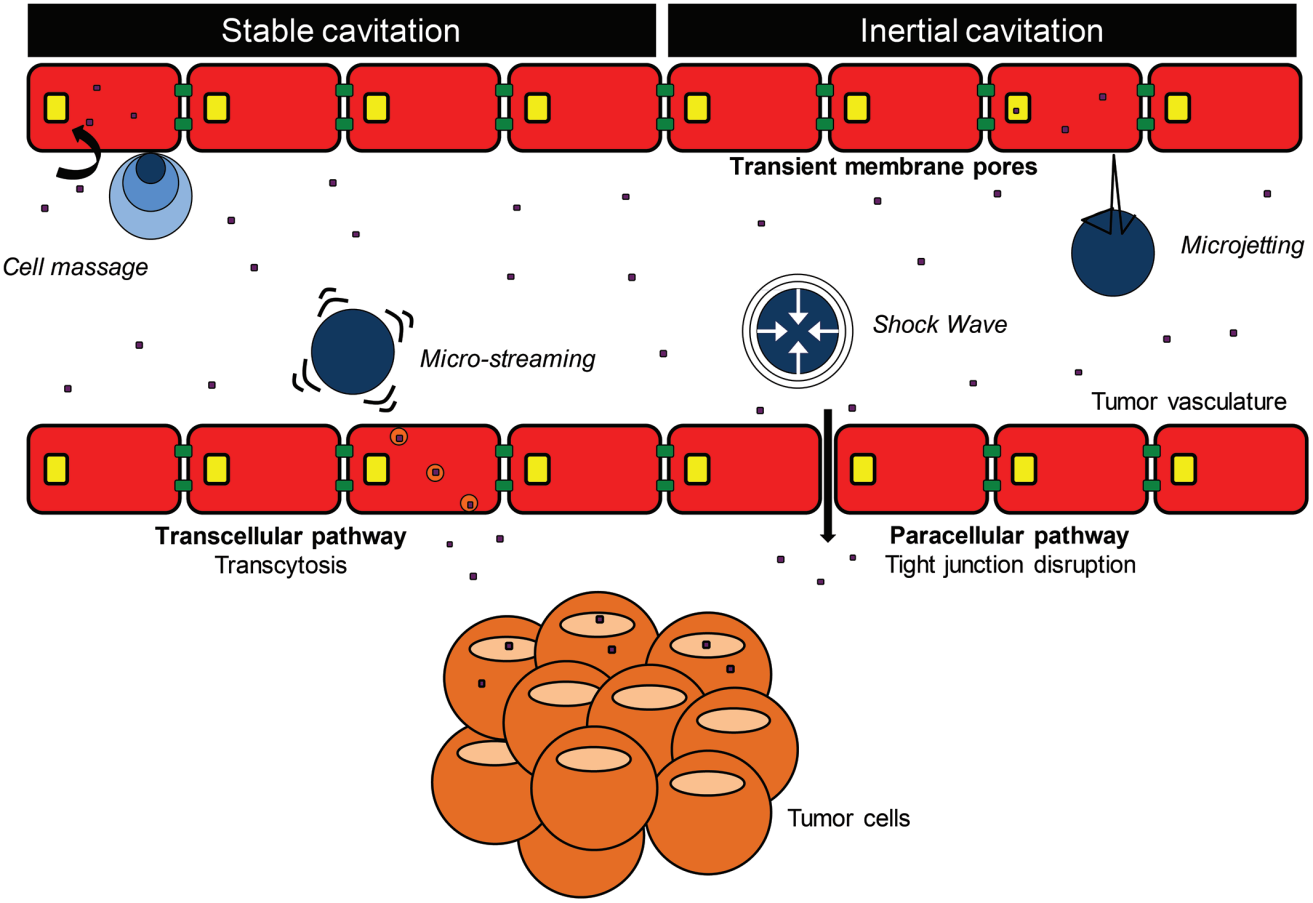
**Extravasation and cellular uptake of drug using microbubble-assisted ultrasound**.

The combination of high frequency ultrasound (1–10 MHz) and ultrasound contrast agents (i.e., consisting of gas microbubbles) was introduced as a promising method in improving the therapeutic efficacy of drugs by increasing local delivery, while minimizing side effects to healthy tissues (Price et al., 1998). In this paper, we refer to this combination as microbubble-assisted ultrasound. The first generation of microbubbles was composed of air encapsulated by albumin (Albunex^®^) or galactose/palmitic acid (Levovist^®^) shells. However, such air-filled microbubbles dissolve in the bloodstream within a few seconds after intravenous (i.v.) administration because of the high solubility of air in blood and their low resistance to arterial pressure gradients. To overcome these issues, a second generation of microbubbles was developed, which were filled with heavy-weight hydrophobic gas (e.g., perfluorocarbon, sulfur hexafluoride) encapsulated by a biocompatible shell (e.g., lipids, polymer; Hernot and Klibanov, 2008; Sirsi and Borden, 2014; **Figure 1A**). In studies on drug delivery by microbubble-assisted ultrasound, the bubbles are mixed with cells *in vitro* or injected *in vivo* intravascularly or directly into the tissue of interest. Microbubble behavior in an ultrasound field has been widely studied, which led to more understanding and subsequent control of the induced bio-effects that can be used for drug delivery (Kooiman et al., 2014). The response of a microbubble to ultrasound waves depends on the acoustic parameters used, such as frequency, pressure levels, and pulse duration. In short, microbubbles stably oscillate over time upon exposure to a low acoustic pressure, a process termed stable cavitation (**Figures 1B** and **2**). These oscillations generate fluid flows surrounding the bubble, known as acoustic micro-streaming, and when in close contact with cells, result in shear stress on the cell membrane, leading to cellular uptake of drugs (Leighton, 1994; Wu, 2002; Doinikov and Bouakaz, 2010). At higher acoustic pressures, microbubbles oscillate more rigorously, leading to their violent collapse and destruction, i.e., inertial cavitation (**Figure 2**). Microbubble disruption can be accompanied by generation of shock waves in the medium close to the microbubbles (Junge et al., 2003; Ohl and Wolfrum, 2003). The ultrasound-induced collapse of the microbubble can be asymmetrical, leading to the formation of high velocity jets (Postema et al., 2005; Ohl et al., 2006). While shock waves induce shear stress to cells in close proximity, resulting in membrane permeability, the high velocity jets can pierce the cell membrane, and thereby create permeability. Stable and inertial cavitation are both exploited to transiently increase the permeability of biological barriers, including the vascular endothelium and plasma membrane, and therefore enhance the extravasation and the cellular uptake of drugs (Lentacker et al., 2014; **Figure 2**).

### Extravasation of Drugs

Microbubbles are intravascular contrast agents, which do not cross the vascular endothelium (Wilson and Burns, 2010). Cavitating microbubbles close to the endothelial wall can result in several bio-effects including vascular disruption, vasoconstriction, or even shutdown of the vessels (Goertz, 2015). Several studies observed that microbubble-assisted ultrasound increased (model-) drug extravasation by stimulating paracellular (i.e., disruption of tight junctions) and transcellular pathways (i.e., transcytosis), both *in vitro* as well as *in vivo* (**Figure 2**; Price et al., 1998; Sheikov et al., 2008; Juffermans et al., 2009; Kooiman et al., 2010). In an *in vitro* endothelial barrier model, Kooiman et al. (2010) showed that microbubble-assisted ultrasound induced a 40% decrease in transendothelial electric resistance showing a loss of endothelial barrier integrity. In addition, Juffermans et al. (2009) showed that microbubble-assisted ultrasound significantly affected the integrity of *in vitro* endothelial monolayers by the destabilization of the tight junctions. At low acoustic pressures (1 MHz, 0.1 MPa), the integrity of the *in vitro* endothelial barrier was restored within 30 min. *In vivo*, an acoustical pressure threshold ranging from 0.1 to 0.75 MPa was required to enhance the extravasation of intravascular agents (e.g., red blood cells, imaging tracers, fluorescent dyes, or drugs) in skeletal muscle (Price et al., 1998), brain (Raymond et al., 2007; Sheikov et al., 2008), liver (Gao et al., 2012), and tumor (Bohmer et al., 2010; Hu et al., 2012) tissues. This extravasation occurs through tight junctions between endothelial cells (0.2–200 μm; Price et al., 1998; Song et al., 2002; Stieger et al., 2007). *In vivo*, the integrity of the blood–brain barrier was restored within 1–4 h following ultrasound exposure (Sheikov et al., 2008; Ting et al., 2012). However, Marty et al. (2012) showed that the duration of extravasation after ultrasound exposure depends on the particle size. The microbubble-assisted ultrasound enhanced transcellular pathways (e.g., transcytosis) have been mainly investigated on the brain vasculature (Raymond et al., 2007; Sheikov et al., 2008; Deng et al., 2012). They reported that low (1 MHz, 0.2 MPa) and high (1.63 MHz, 1-3 MPa) acoustic pressures increased the number of transcytotic vesicles on both the luminal and abluminal surface of the endothelium. Sheikov et al. (2004) hypothesized that the transient vasoconstriction constitutes a potential cause for the increased transcytosis *in vivo*. In addition, Hu et al. (2012) showed that the destruction of microbubbles with a high acoustic pressure (5 MHz, 2 MPa) decreased the tumor blood flow for 30 min before it returned back to normal, without an increase in hemorrhage. Whereas it was demonstrated that the extravasation of fluorescent dextrans was enhanced during this period, the authors did not investigate whether transcytosis was involved. Transient vasoconstriction has been only reported in mice, which exhibit higher vasomotor excitability than other rodents and animal species.

#### Heating and Acoustic Radiation Force

**FIGURE 3:**
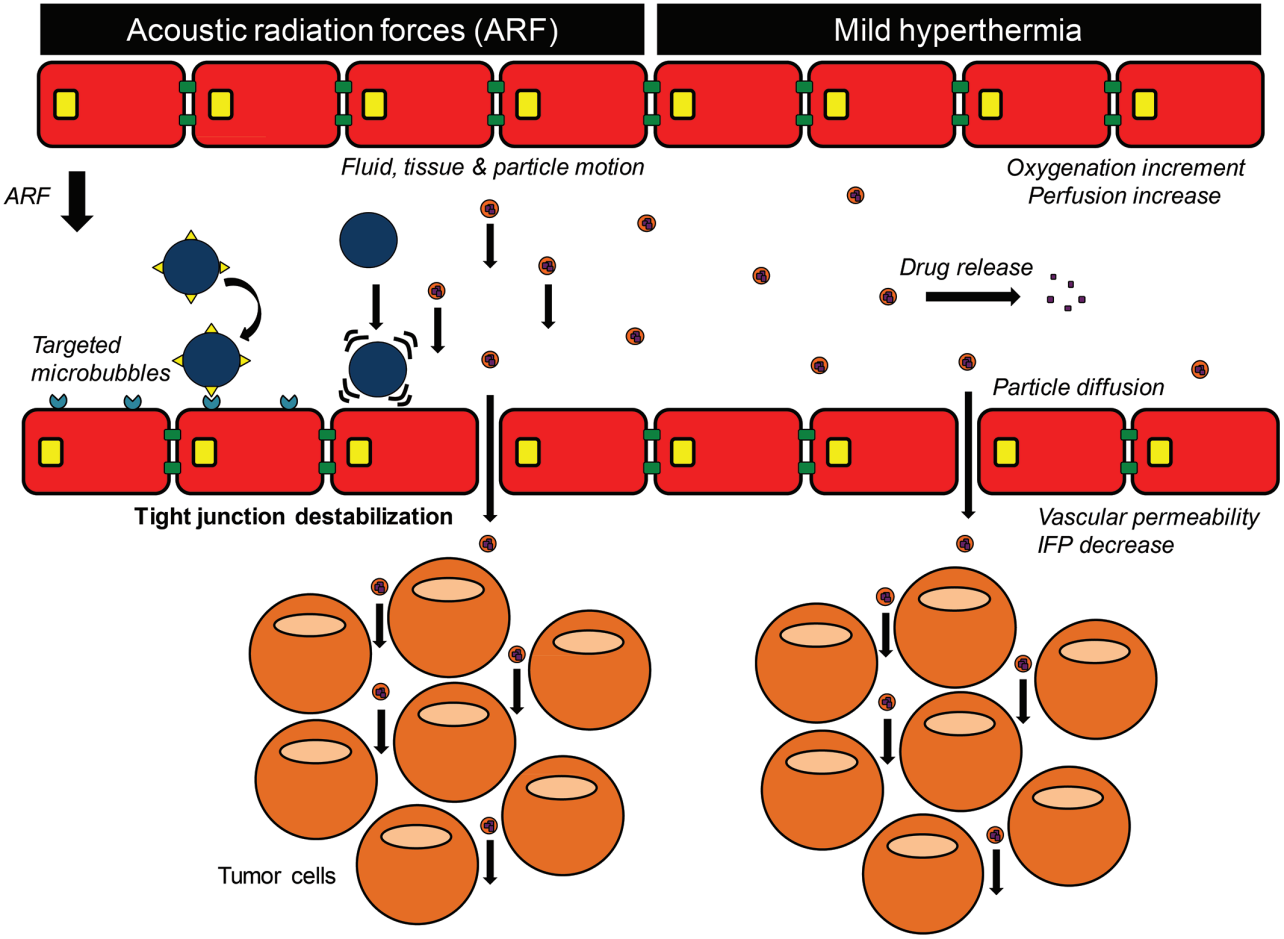
**Acoustic radiation forces and ultrasound-induced hyperthermia**.

Besides cavitation, ultrasound can also induce heating and acoustic radiation force (ARF) to improve the extravasation of drugs (Deckers and Moonen, 2010). Heating can result from the absorbance of acoustic energy as the ultrasound beam propagates through tissue. Mild heating of a tumor (41 – 43°C for 10 – 60 min) may improve the therapeutic efficacy of drugs by acting on tumor hemodynamics (**Figure 3**): (i) by increasing tumor perfusion, thus enhancing drug bioavailability in tumor tissue (Song, 1984); (ii) by increasing vascular permeability (Lefor et al., 1985; Kong et al., 2001) and reducing tumor interstitial pressure (Vaupel and Kelleher, 2012), leading to better drug penetration within tumor tissue. In addition, local heating can act as an external trigger for drug release from a carrier, e.g., thermosensitive nanoparticles (Yatvin et al., 1978; Lindner et al., 2004; Manzoor et al., 2012; Hijnen et al., 2014; Al Sabbagh et al., 2015). Ultrasound can also generate directional ARF on molecules along its propagation path (Sarvazyan et al., 2010; **Figure 3**). This enhances the extravasation of free drug or drug-loaded nanoparticles into tumor tissue by causing tissue shear stress and opening of endothelial tight junctions (Seidl et al., 1994; Mesiwala et al., 2002). ARF induces fluid streaming through the interstitium, thus improving biodistribution of intravascular dyes and drugs in the target tissue (Lum et al., 2006; Hancock et al., 2009). Using optical imaging, Shortencarier et al. (2004) showed that the application of ARF induced visible aggregates of fluorescent dye-loaded gas lipospheres in the direction of the beam on the far vessel wall. The lipospheres disappeared when the ARF pulses were turned off (Shortencarier et al., 2004). In addition to lipospheres, ARFs can push circulating microbubbles toward the endothelial wall, thereby improving microbubble–cell contact, which might enhance cavitation-mediated extravasation of intravascular compounds (Rychak et al., 2005; Wang et al., 2014). Using ultrasound imaging, Frinking et al. (2012) reported that ARF (38 kPa PNP, 95% DC) induced a sevenfold increase in the binding of VEGFR2-targeted microbubbles (also known as BR-55) on the endothelial wall in a prostate adenocarcinoma rat model compared with the binding without ARF.

### Cellular Uptake of Drugs

Cavitating microbubbles in the vicinity of the plasma membrane can result in cell permeabilization by creating membrane pores and stimulating the endocytosis pathways, thereby facilitating intracellular drug uptake. Based on the uptake or release of non-permeant dyes (Meijering et al., 2009; Kaddur et al., 2010) and by measuring changes in membrane electrophysiology (Tran et al., 2007; Juffermans et al., 2008), previous studies showed that microbubble-assisted ultrasound induced a transient increase in membrane permeability through the generation of transient hydrophilic pores. The intracellular delivery of molecules through membrane pores is likely governed by passive diffusion or by ultrasound-mediated propulsion (i.e., microstreaming, ARF; Shortencarier et al., 2004; Lum et al., 2006). The size of these ultrasound induced pores depend on the acoustic parameters used, ranging from 1 to 94 nm at 0.19 MPa PSP and from 2 to 4 μm at 0.48 MPa PSP (Yang et al., 2008).

In addition to hydrophilic pore formation, enhancement of endocytosis has also been demonstrated following microbubble-assisted ultrasound exposure (Meijering et al., 2009). Electrophysiological studies reported that microbubble-assisted ultrasound induced an influx of Ca^2+^, followed by an activation of BK_Ca_ channels that results in local hyperpolarization of the cell membrane (Tran et al., 2007; Juffermans et al., 2008). At moderate ultrasound conditions (1 MHz, 0.15–0.3 MPa), the membrane hyperpolarization facilitates the molecular uptake through endocytosis and macropinocytosis. Similar to pore formation, the contribution of endocytosis processes depends strongly on the marker size and the acoustic pressures. Meijering et al. (2009) reported that low acoustic pressures (1 MHz, 0.22 MPa PNP) resulted in the cellular uptake of 4.4 and 70 kDa fluorescent dextrans through membrane pores while the entrance of 155 and 500 kDa fluorescent dextrans is dominated by endocytosis pathways. It should be mentioned that little is known about the faith of the agents in the endocytic vesicles, if they are degraded in the lysosome or escape from the endosome. However, De Cock et al. (2015) showed that increasing the acoustic pressures (1 MHz, 0.5 MPa, PNP) induced the intracellular delivery of large fluorescent dextrans (2 MDa) to shift from uptake by endocytosis to uptake via the membrane pores. Regardless of the mechanism of uptake, the duration of microbubble-assisted ultrasound-mediated uptake is dependent on the plasma membrane recovery time, which is a few seconds to a few hours (van Wamel et al., 2006; Lammertink et al., 2015). The different kinetics depends on the ultrasound conditions, the model drug size and the cell physiology.

## Anti-Cancer Drug Delivery Protocols

As any drug delivery technique, microbubble-assisted ultrasound treatments aim to deliver optimal quantities of chemotherapeutic drugs in targeted tumor cells and tissues. The efficiency of this delivery method depends on (i) sufficient accumulation of microbubbles and drugs near tumor cells or tissues, which is directly influenced by the properties of microbubbles, drugs (i.e., plasma circulation lifetime), and tumor (i.e., vascularization, localization), as well as administration routes (i.e., intratumoral, intravenous, intraperitoneal); (ii) the acoustic conditions including ultrasound parameters (i.e., central frequency, acoustic pressure, exposure time, etc.) and devices (i.e., home-made, commercial, medical systems); (iii) treatment schedule including the time interval between the drug and/or microbubbles administration and ultrasound treatment as well as the number of microbubble-assisted ultrasound drug delivery treatments and the time interval between them. Over the past decade, the influence of these factors on drug delivery efficiency has been investigated in order to enhance the intratumoral (i.t.) accumulation of drug, thereby increasing the treatment effect, while minimizing side effects to healthy tissues. This review shows that the drug delivery efficacy varied between the tumor models used *in vivo*. It is commonly known in the field that the tumor type is an important determinant for successful drug delivery. This is due to the specific properties of each tumor tissue, such as differences in tissue organization, extracellular matrix, presence of necrosis and hypoxia, cell density, and the endothelial lining of the tumor vasculature (Chauhan et al., 2011). To the best of our knowledge, no comparative study between tumor tissues with different properties has been reported using microbubble-assisted ultrasound for drug delivery. However, unlike many other drug delivery strategies, sonochemotherapy does not depend on the enhanced permeability and retention (EPR) effect, which is very heterogeneous between or within tumors, and often overestimated (Lammers et al., 2012). Interestingly, you could argue that the largest effect of sonochemotherapy can be expected in tissues with ‘non-leaky’ vessels, such as the brain (Ting et al., 2012), since the potential of increasing extravasation is highest. An overview of different drug delivery protocols and outcomes *in vitro* and *in vivo* are shown in **Tables 1** and **2**, respectively. It should be noted that this is not a complete overview, but rather a selection of different drug delivery protocols.

### Microbubbles

In most studies, clinically approved microbubbles (i.e., SonoVue^®^, Definity^®^) for ultrasound imaging are employed for drug delivery. The use of these microbubbles may facilitate the clinical translation of sonochemotherapy, but any undesired side effect might have a negative impact on the use of these microbubbles in ultrasound-based diagnostics. Modification of these microbubbles (e.g., drug-loaded microbubbles) for therapeutic applications will delay clinical translation, requiring new authorization from the regulatory and health authorities.

#### Coadministration of Microbubbles and Drug

The simplest method for drug delivery using microbubble-assisted ultrasound is to use coadministration (Heath et al., 2012; Unga and Hashida, 2014). This approach includes drugs that are administered in patients anyway in current clinical practice, with the addition of an injection of (clinically approved) microbubbles. Microbubbles and drugs can be mixed in solution *in vitro* and the mixture is then injected *in vivo.* This strategy offers two main advantages: (i) both constituents can be handled completely separately until *in vitro* or *in vivo* administration; (ii) instead of mixing microbubbles and drug before injection, two separate injections of the constituents can also be performed, thus allowing drugs to reach plasma peak levels before injecting microbubbles (Escoffre et al., 2013b). Microbubbles have a short circulation time and therefore need to be exposed to ultrasound within minutes after injection, otherwise they will be degraded and unable to induce bio-effects. The coadministration approach seems to be the best strategy for *in vitro* purposes (Escoffre et al., 2011; Sorace et al., 2012) or, *in vivo*, i.t. injection of the mixture (Sasaki et al., 2014), where similar spatio-temporal distribution of both components will be ensured. Iwanaga et al. (2007) showed that the *in vitro* delivery of bleomycin using microbubble-assisted ultrasound induced twofold decrease in cell viability compared to the bleomycin treatment alone (**Table 1**). *In vivo*, they reported that the exposure of a tumor to ultrasound following the i.t. co-injection of microbubbles and bleomycin also resulted in a twofold decrease in tumor volume (Iwanaga et al., 2007). Kotopoulis et al. (2014) coadministered commercially available microbubbles and gemcitabine i.v. in a pancreatic cancer model in mice. They showed that ultrasound exposure (1 MHz, 0.2 MPa PNP) decreased the tumor volume twofold compared to gemcitabine alone (Kotopoulis et al., 2014). Opposed to the advantages of coadministration using clinically approved microbubbles and drugs that allow clinical translation, there are also disadvantages. The main limitations of the i.v. injection of microbubble/drug mixture compared to drug-loaded microbubbles are: (i) differential distribution of both constituents because of their physicochemical properties; (ii) fast degradation of free drugs and microbubbles; (iii) unspecific accumulation of free drugs in the healthy tissues.

#### Drug-Loaded Microbubbles

To overcome these limitations of i.v. coadministration, microbubbles have been modified to function not only as cavitation nuclei, but also as drug delivery carriers. For example, lipophilic drugs can be incorporated into the lipid monolayer shell of microbubbles or dissolved in an oil pocket between the gas core and the microbubble’s shell (Ibsen et al., 2013). By applying this approach, Burke et al. (2014) found that the application of ultrasound (1 MHz, 1.2 MPa, every 5 s for 60 min) on subcutaneous C6 glioma tumor following the i.v. injection of 5-FU-loaded microbubbles (1 × 10^5^ microbubbles/g body weight) led to twofold decrease in tumor volume compared to 5-FU treatment alone (Burke et al., 2014). While these approaches seem to be promising, the low drug loading capacity of microbubbles is a major drawback. Consequently, the use of drug-loaded microbubbles requires either enhancement of the drug loading efficiency, administration of high dose of drug-loaded microbubbles, or application of consecutive treatments.

The small size of microbubbles and their gaseous lumen restricts the space for drug loading. Recent publications reported that the binding of drug-loaded nanoparticles on the microbubble’s surface could increase the amount of loaded drug (Geers et al., 2011). The loading efficiency can be further improved by applying multiple layers of drug-loaded nanoparticles around the microbubble shell. The binding of drug-loaded nanoparticles on microbubbles may not be necessary for polymer-based microbubbles, as significant amounts of (model) drug can be loaded into the polymer-based shell (Fokong et al., 2012). Cochran et al. (2011) showed that the loading capacity is higher for hydrophobic drugs compared to hydrophilic drugs, and that the acoustic properties of the microbubbles were unaffected (Cochran et al., 2011).

Based on current studies, a high dose of drug-loaded microbubbles, i.e., >10^10^ microbubbles, must be intravenously injected to reach a therapeutic dose similar to the one used in clinical chemotherapy. However, the recommended diagnostic doses of microbubbles currently approved for contrast-enhanced ultrasound imaging (e.g., SonoVue^®^, Definity^®^) are between 10^9^ and 10^10^ microbubbles for an 80-kg adult (Wilson and Burns, 2010). Nevertheless, preclinical and clinical studies have reported a good tolerance with 100- and 1000-fold higher doses of these microbubbles in non-human primates and patients (Grauer et al., 1996; Bokor et al., 2001). Consequently, the injection of a high dose of drug-loaded microbubbles may not be a limitation for clinical use, but further preclinical studies might be necessary to identify any potential toxicity of high concentrations of liposome and shell’s components (i.e., lipid, polymer, and albumin).

Finally, several preclinical studies reported the use of repeated sonochemotherapy treatments (Kang et al., 2010; Tinkov et al., 2010; Li et al., 2012; Ting et al., 2012). For example, Li et al. (2012) reported that the repetitive treatment (i.e., once a day for seven consecutive days) of subcutaneous hepatic tumor using 10-hydroxycamptothecin-loaded microbubbles (4 mg/kg) induced twofold stronger decrease in tumor volume in a subcutaneous hepatic tumor model (1 MHz, 2 W/cm^2^, 6 min) compared to the 10-hydroxycamptothecin-based chemotherapy alone (Li et al., 2012).

#### Targeted Microbubbles

**FIGURE 4:**
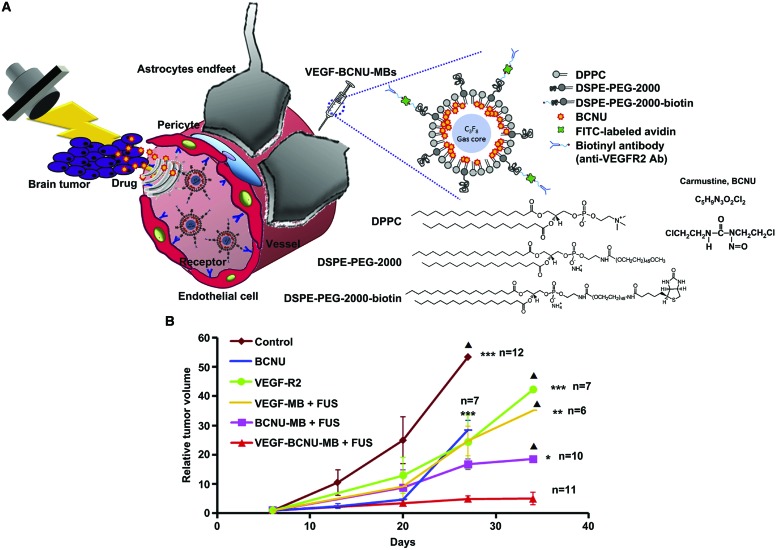
**Intracerebral BCNU delivery using VEGFR2-targeted and BCNU-loaded microbubbles with focused ultrasound for the glioma treatment (Adapted with permission from Fan et al., 2013 – Copyright © 2012 Elsevier Ltd.). (A)** Antiangiogenic-targeting BCNU-loaded microbubbles combined with focused ultrasound for glioma treatment. **(B)** Tumor growth curve. BCNU, Carmustine; VEGF-R2, anti-angiogenic antibody; VEGF-MB, VEGF-targeting microbubbles; BCNU-MB, BCNU-loaded microbubbles; VEGF-BCNU-MB, VEGF-targeting BCNU-loaded microbubbles; FUS, focused ultrasound. ^∗^*p* < 0.05; ^∗∗^*p* < 0.01; ^∗∗∗^*p* < 0.001. Solid triangle, less than 3 rats were presented.

Microbubbles can be modified to target specific overexpressed markers on tumor cells (i.e., PSMA, prostate specific membrane antigen; LHR, luteinizing hormone receptor) or tumor microvasculature (VEGF-R2, vascular endothelial growth factor receptor -2) through attachment of targeting ligands or antibodies onto the microbubble’s shell (Kiessling et al., 2012, 2014; Novell et al., 2013). This may lead to enhanced accumulation of the microbubbles in the target tumor cells or tissues. For example, Fan et al. (2013) designed targeted BCNU-loaded microbubbles, which bind the VEGF-R2 overexpressed on tumor microvasculature (VEGFR2-BCNU-loaded microbubbles; **Figure 4A**). The exposure of orthotopic glioma to ultrasound (1 MHz, 0.7 MPa, 1 min/sonication site) following i.v. injection of VEGFR2-BCNU-loaded microbubbles (1.25 mg BCNU) resulted in 1.75-fold decrease in tumor volume compared to the untargeted BCNU-loaded microbubbles (**Figure 4B**; Fan et al., 2013). The use of microbubbles targeting overexpressed markers on the tumor cells themselves is limited to *in vitro* drug delivery, i.t. or intraperitoneal (i.p.) injection of microbubbles and drugs, primarily because the microbubbles, when administrated intravenously, cannot extravasate due to the size (Cavalieri et al., 2010). For imaging, several groups have reported on the *in vivo* accumulation of targeted microbubbles in the tumor microvasculature by binding inflammation markers overexpressed on tumor endothelial cells (Deshpande et al., 2010). Although these microbubbles were designed as ultrasound contrast agents for molecular imaging, it might be possible to develop optimal tissue- or organ-selective drug delivery agents by combining targeting capacities and drug loading of microbubbles (Kiessling et al., 2012). However, no evidence of their use for drug delivery has been reported yet.

To summarize, the coadministration of drugs/microbubbles and drug-loaded microbubbles can both be used for drug delivery. The coadministration approach is likely to be the fastest way into the clinic, as it combines clinically approved drugs and microbubbles. However, the drug-loaded microbubbles may hold the greatest therapeutic potential, as it locally releases the drug upon ultrasound exposure. Since this approach represents new therapeutic entities, such ‘therapeutic microbubbles’ require extensive testing for safety and efficacy before they can be approved for clinical use. To the best of our knowledge, no study has been published that directly compares drug-loaded microbubbles with coadministration of free drugs and microbubbles at equal dosing schemes.

### Administration Routes

The most direct administration route for drug delivery is i.t. injection (Sonoda et al., 2007; Sasaki et al., 2014). The advantages of i.t. administration over systemic injection include the circumvention of the transvascular barrier and the generation of transient interstitial pressure gradients. The latter can induce convection and tissue deformation, which can decrease the connectedness of the extracellular matrix and size of pores in the tumor interstitial space (Frenkel, 2008). By using i.t. administration, a high drug dose can be directly delivered into the target tumor while minimizing its side effects toward healthy tissues. This administration route overcomes the drawback related to the short plasma half-life of drugs and microbubbles after i.v. injection. In addition, this route is most interesting for hydrophilic small chemotherapeutic drugs that have difficulties to enter tumor cells. By applying i.t. injection, microbubbles and drugs are distributed within the tumor by diffusion and convection, and subsequent US exposure will result in drug uptake in tumor cells. However, in i.t injection, there are some limitations such as the injected volume and the accessibility of the tumor site, which restrict the application of microbubble-assisted ultrasound to superficial tumors such as melanoma, and cutaneous and subcutaneous tumors.

For deep-seated tumors, most protocols recommend injection of drugs and microbubbles via blood flow, providing better access to deeper tumors (Treat et al., 2012; Yan et al., 2013; Burke et al., 2014). The i.v. route is a relatively easy and safe way to be used in the clinic for the administration of therapeutics and microbubbles. As previously described, the main limitation of this administration route is the rapid clearance of drug from plasma and the unspecific accumulation of this drug in healthy tissues. Therefore, drugs can be loaded on microbubbles to overcome these shortcomings (Ting et al., 2012; Sirsi and Borden, 2014). The success of i.v. drug delivery relies on sufficient tumor vascularization, thus restricting the application of this administration route to hypervascularized tumors. Next to extravasation, microbubble-assisted ultrasound can also increase the penetration of drugs into the tissue. In addition, it can “homogenize” drug uptake, since drug distribution tends to be very heterogeneous throughout the tumor. Since microbubble-assisted ultrasound will mostly affect the vascular endothelium, the i.v. route is most suitable for drugs that can benefit from ultrasound-induced extravasation and penetration or intracellular delivery in endothelial cells.

Recent studies reported that the i.p. injection may be useful for drug delivery using microbubble-assisted ultrasound for the treatments of primary peritoneal cancers or cancers with i.p. metastases. Pu et al. (2014) investigated the i.p. delivery of paclitaxel (PTX) for the treatment of ovarian cancer using luteinizing hormone-releasing hormone analog (LHRHa) -targeted and PTX-loaded microbubbles (20 mg/kg PTX) and ultrasound (0.3 MHz, 1 W/cm^2^, 3 min). This therapeutic protocol led to a twofold increase in apoptotic index and a 2.5-fold decrease in vessel number compared to the single injection of free PTX or PTX delivery using ultrasound alone (Pu et al., 2014). Due to the microbubble size, penetration of the microbubbles by convection throughout the tumor is hindered, thereby limiting the tumor cell binding to the peripheral rim of the tumor. Nevertheless, the targeted microbubbles in this study showed superior efficacy compared to the untargeted bubbles.

### Ultrasound Devices, Transducer, and Parameters

Several investigations showed extensive optimization of the acoustic parameters to result in an efficient and safe *in vitro* and *in vivo* drug delivery. Among these studies, clinical ultrasound scanners have been used to deliver drugs using microbubble-assisted ultrasound (Tinkov et al., 2010; Sasaki et al., 2014), which has the advantage of enabling both imaging of- and drug delivery to the targeted tumor. However, the ultrasound settings that are allowed on such equipment are limited for safety reasons. Specific ultrasound parameters [low cycles and mechanical index (MI) 0.5 < MI < 1.9] are used to destroy microbubbles during a diagnostic tissue perfusion study (Szabo, 2013). However, such parameters might not be efficient for drug delivery. In addition, clinical ultrasound probes are unfocused and thus the ultrasound energy will have substantial effects in the regions surrounding the target tissue. Clinical ultrasound scanners are “black-boxes” which do not allow controlling all ultrasound parameters. Hence, home-made and commercial therapeutic ultrasound devices have been designed to control many ultrasound parameters, which can subsequently be optimized for drug delivery (Zhao et al., 2011; Lin et al., 2012; Escoffre et al., 2013a). Ultrasound transducers used in the literature can be focused or unfocused (Sanches et al., 2011). Focused beams are created using spherically curved transducers, which greatly increase the ultrasound intensity in a small region of interest, e.g., a tumor. Due to a lack of standardized calibration methods concerning the applied ultrasound parameters and the heterogeneity in equipment used, it is not straightforward to compare the results of most studies directly (ter Haar et al., 2011).

The transmission center frequency used for *in vivo* drug delivery studies listed in **Tables 1** and **2** ranges from 0.3 to 2.25 MHz. The choice of frequency to be used can depend on the microbubble’s size and its resonance frequency, but also on the depth of the tissue to be reached, as higher frequencies suffer from increased attenuation. The resonance frequency of microbubble decreases as their size increases (Minnaert, 1933). When using a low frequency range, the acoustic pressure threshold to initiate microbubble cavitation can be reduced, thereby limiting putative tissue damage. In most of the reported investigations, 1 MHz was used as a frequency to achieve drug delivery using microbubble-assisted ultrasound (**Tables 1** and **2**).

The ultrasound dose is usually expressed in different units depending on whether a medical ultrasound scanner, commercial or laboratory-made device is used for drug delivery (**Table 2**). With home-made or commercial therapeutic ultrasound devices, ultrasound exposure is usually expressed either in acoustic pressure amplitude (kPa) or in intensity (W/cm^2^) while for medical ultrasound scanners, the dose is usually expressed in the terms of MI (expressed as the ratio of the peak negative pressure in MPa to the square root of the frequency in MHz). Among the published studies, it is not clearly stated whether ultrasound intensity are spatial averaged, temporal averaged intensity (I_SATA_) or spatial peak, temporal averaged intensity (I_SPTA_). I_SATA_ is frequently used when non-focused transducer is employed for drug delivery. Ultrasound intensities ranging from 0.064 to 3 W/cm^2^ (n.b., I_SPTA_ 0.0003 – 0.9 W/cm^2^ for ultrasound-based diagnostics) have been applied in recent studies to deliver drugs in tumor tissue without injuries (Kang et al., 2010; Lu et al., 2011). The MI used for *in vivo* drug delivery ranges from 0.2 to 2 (n.b., MI threshold for clinical diagnosis is 1.9). Drug delivery requires a minimum MI known as the permeabilization threshold, which is typically lower than 1 (Choi et al., 2007). Exposure of tumor tissues above, but near the cavitation threshold has so far yielded the most promising results of drug delivery without significant side effects. Increasing the ultrasound dose further enhanced drug delivery in the target tissue but was also accompanied by hemorrhage and tissue injuries (Kang et al., 2010; Lu et al., 2011).

The duty cycle is the percentage of time that an ultrasound device is transmitting acoustic waves. The duty cycle ranges from 0.25 to 50% for drug delivery into tumors (**Table 2**). To prevent thermal tissue damage, low duty cycles are used when high ultrasound intensities are applied and vice versa (Lin et al., 2012; Wei et al., 2013).

Ultrasound exposure time plays a major role in drug delivery using microbubble-assisted ultrasound. During this time, ultrasound pulses are emitted repeatedly at a pulsing interval to induce the complete destruction of microbubbles in the targeted tumor. Ultrasound exposure times from 2 s to 10 min have been reported (**Table 2**). However, exposure times of 1–5 min are recommended to prevent tissue injuries (e.g., hemorrhages; Mei et al., 2009; Yan et al., 2013).

### Treatment Schedule

The therapeutic protocol depends on the duration of microbubble-assisted ultrasound-mediated permeability of tumor tissues and the pharmacokinetics of chemotherapeutic drugs. Some studies reported drug administration at different time points following the exposure of tumor to microbubble-assisted ultrasound to assess the duration of enhanced permeability (few seconds – few hours, depending on the particle size; Marty et al., 2012; Tzu-Yin et al., 2014; Lammertink et al., 2015). Other investigations recommend waiting for the peak concentration of drug in the blood before the administration of microbubbles and the subsequent exposure of tumors to ultrasound. For example, Escoffre et al. (2013b) succeeded to optimize therapeutic efficacy of irinotecan using microbubble-assisted ultrasound in subcutaneous glioblastoma. In this study, the protocol consisted of an i.v. injection of irinotecan followed 1 h later by an i.v. administration of microbubbles (Escoffre et al., 2013b). This delay is required to reach the maximal systemic concentration of SN-38, the active metabolite of irinotecan, in the blood. This strategy induced a twofold decrease in tumor volume and perfusion compared to irinotecan without subsequent ultrasound exposure.

In most therapeutic protocols using the coadministration approach or drug-loaded microbubbles, ultrasound was applied to the tumors immediately (5–10 s) after microbubble injection (Sonoda et al., 2007; Matsuo et al., 2011). This strategy supposes that drugs and microbubbles are sufficiently accumulated in the target tissue during the few seconds following their administration. However, no real evidence has been reported whether this is actually the case. In addition, monitoring of microbubble arrival at the target tissue using contrast-enhanced ultrasound prior to ultrasound therapy is rarely performed. At present, all investigations show that at least several consecutive treatments (2–20 times) at optimal time intervals (1 day – 1 week) are required to achieve significant decrease in tumor growth or even tumor eradication (**Table 2**).

## Therapeutic Efficacy vs. Safety: from *In Vitro* to Preclinical Studies

**FIGURE 5:**
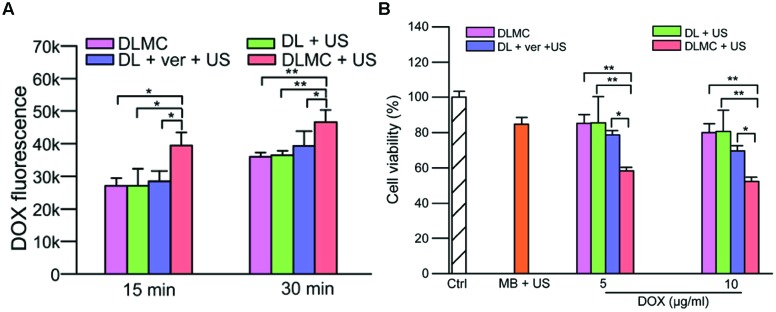
**(A)** Intracellular doxorubicin (DOX) concentration in MCF-7/ADR cells 15 and 30 min post treatment. **(B)** Cell cytotoxicity after several treatments with or without US. DLMC, DOX-liposome-microbubble complexes; DL, DOX-liposomes; ver, verapamil; US, Ultrasound. ^∗^*p* < 0.05, ^∗∗^*p* < 0.01 (Adapted with permission from Deng et al., 2014 – Copyright © 2014 Elsevier Ltd.).

As described above, the therapeutic benefit of drug delivery using microbubble-assisted ultrasound relies on enhancing accumulation of drugs in tumor cells or tissues and on decreasing their deposition in healthy tissues, thus reducing their side effects (Tinkov et al., 2010; Li et al., 2012; Fan et al., 2013; Burke et al., 2014). Using the coadministration approach or drug-loaded microbubbles, microbubble-assisted ultrasound enhances *in vitro* the therapeutic efficacy of clinically approved chemotherapeutics including doxorubicin (Dox), cisplatin, bleomycin, PTX, and docetaxel (**Table 1**). Most *in vitro* studies only monitor drug effectiveness with or without microbubble-assisted ultrasound. However, some studies also investigated the underlying mechanism. For example, Deng et al. (2014) showed enhanced intracellular Dox levels (**Figure 5A**) and increased retention due to a down-regulation of P-glycoprotein following ultrasound exposure in the presence of Dox-liposome loaded microbubbles. This resulted in a significant increase of double-stranded DNA breaks and reduced cell viability (**Figure 5B**). The exposure of tumor cells to microbubble-assisted ultrasound without any drugs had no or few effects on cell viability (>85% cell viability).

In *in vivo* studies it was clearly observed that microbubble-assisted ultrasound improves the therapeutic efficacy of drugs for different tumor animal models. However, most studies only monitor outcomes like survival and tumor size. Unfortunately, i.t. drug accumulation and distribution is often not investigated. Regardless of the administration route, only 40% of preclinical studies showed that an enhanced therapeutic efficacy could be attributed to increased i.t. drug levels. For example, Tinkov et al. (2010) demonstrated that the exposure of pancreas carcinoma in rats to ultrasound (1.3 MHz, 1.2 MPa PNP, four frames of ultrasound every four cardiac cycles) after i.v. injection of DOX-loaded microbubbles (140 μg – 3.14 × 10^9^ microbubbles) induced a 10-fold increase in i.t. DOX accumulation compared to DOX-loaded microbubble injection alone (Tinkov et al., 2010). This therapeutic protocol led to a twofold decrease in tumor volume.

**FIGURE 6:**
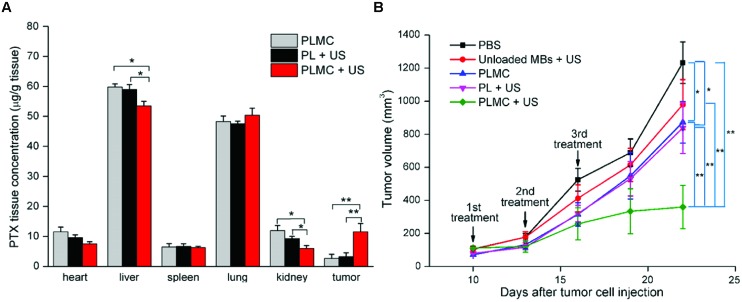
**Paclitaxel (PTX) delivery by PTX-loaded microbubble with ultrasound for breast cancer treatment (Adapted with permission from Yan et al., 2013 – Copyright © 2013 Elsevier Ltd.). (A)** Paclitaxel *in vivo* distribution in heart, liver, spleen, lung, kidney and tumors 1 h after injection of paclitaxel-loaded microbubble complexes (PLMC) alone, paclitaxel liposomes (PL) + US or PLMC + US; **(B)** *In vivo* growth inhibition in 4T1-tumor bearing mice within 22 days. Mice were treated with PBS (squares), unloaded microbubbles + US (circles), PLMC without US (upward triangles), PL + US (downward triangles) or PLMC + US (diamonds) on days 10, 13 and 16 after tumor cell injection. Results represent mean ± SD, *n* = 6. ^∗^*p* < 0.05; ^∗∗^*p* < 0.01.

Next to increased drug concentration in the target tissue, one of the expected consequences of i.t. drug delivery using microbubble-assisted ultrasound is the reduction of drug deposition in healthy tissues. However, this effect is expected to be only significant for local release from drug-loaded microbubbles compared to the coadministration approach, where free drugs can enter healthy tissue anyway, without ultrasound exposure. Less than 10% of preclinical studies reported on drug distribution toward healthy tissues. Among the studies that do measure this, Yan et al. (2013) reported that the application of ultrasound (2.25 MHz, 1.9 MPa, 10 min, three treatments: one treatment every 3 days) on subcutaneous breast tumor following the i.v. injection of PTX-loaded microbubbles (120 μg – 1 × 10^9^ microbubbles) resulted in fourfold increase in i.t. accumulation of PTX (**Figure 6A**) and 2.5-fold decrease in tumor volume compared to PTX-loaded microbubbles treatment alone (**Figure 6B**). The authors also investigated the drug biodistribution in healthy organs including heart, liver, spleen, lung, and kidney 1 h after i.v. administration of the PTX-loaded microbubbles and ultrasound exposure (Yan et al., 2013). The PTX biodistribution in heart, spleen, and lung was not significantly different between mice that received PTX-loaded microbubbles treatment alone or combined with ultrasound (**Figure 6A**). However, the PTX delivery using microbubble-assisted ultrasound led to a slight but significant decrease in PTX concentration in liver and kidney compared to PTX-loaded microbubbles injection alone (**Figure 6A**). No significant loss of body weight and other adverse effects were observed during the therapeutic procedure. Moreover, Ting et al. (2012) designed a therapeutic protocol based on BCNU-loaded microbubbles (0.8 mg – 1 × 10^10^) with focused ultrasound (1 MHz, 0.5–0.7 MPa, 2 sonications, 1 min/sonication) to improve BCNU-based chemotherapy for glioblastoma treatment. They showed that the encapsulation of BCNU in microbubbles prolonged its circulatory half-life fivefold and intrahepatic accumulation of BCNU was reduced fivefold due to the slow reticuloendothelial system uptake of BCNU-loaded microbubbles (Ting et al., 2012). These microbubbles alone or in combination with focused ultrasound were associated with lower levels of aspartate- and alanine-aminotransferases compared to free BCNU, suggesting that these microbubbles may effectively reduce liver toxicity and damage. In glioblastoma-bearing rats, BCNU-loaded microbubbles with ultrasound led to 13-fold decrease in tumor volume. However, median survival was extended by only 12% compared to BCNU and control.

However, for all microbubble-based ultrasound therapies, the effect on the vasculature should be closely monitored. There is a ‘fine line’ between stimulating vascular permeability and inducing vascular damage, which can result in inhibition of tumor perfusion. Although this may be a desired effect in some studies, for drug delivery from the vasculature, a reduced tumor perfusion might limit the i.t. drug supply. For example, Burke et al. (2011) demonstrated that the mechanical effect of low duty cycle ultrasound (1 MHz, 1 MPa PNP) in combination with microbubbles could inhibit glioma growth by blocking tumor perfusion. The anti-vascular action of microbubble-assisted ultrasound (1 MHz, 1.6 MPa PNP) was also adopted by Todorova et al. (2013) who subsequently injected an anti-angiogenic agent to prevent the formation of new vessels. In the light of these results, animal studies conducted with ultrasound pressures >1.0 MPa should always include a control group with microbubble-assisted ultrasound only, and preferably monitor the perfusion of the exposed tissue (e.g., by Doppler or contrast-enhanced ultrasound imaging).

To summarize, a growing number of preclinical investigations show promising results for future clinical applications. Future studies will have to confirm that the increase in therapeutic efficacy of sonochemotherapy is correlated with enhanced i.t. accumulation and penetration of drugs. To demonstrate the safety of this method, drug biodistribution toward healthy organs and tissues should be monitored and physiological functions of healthy organs should be examined using imaging, histological analysis, and blood biochemistry analysis. Information on *in vivo* biodistribution and pharmacokinetics of intact and destroyed microbubbles as well as an evaluation of their systemic side effects are still absent in most available publications. These aspects need to be integrated in future studies. It must be noted that the sonochemotherapy approach has mainly been evaluated in small animals. Studies in large animals are still lacking and might face challenging and unexpected physical (e.g., ultrasound penetration depth, ultrasound attenuation) and biological (e.g., plasma life time of drug and microbubbles) limitations.

## Translation to the Clinics

**FIGURE 7:**
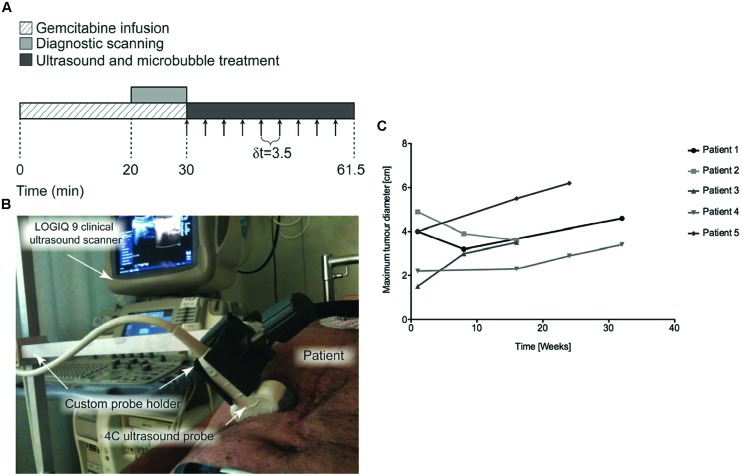
**Treatment of human pancreatic adenocarcinoma using gemcitabine using microbubble-assisted ultrasound (Adapted with permission from Kotopoulis et al., 2013 – Copyright © 2013 *Am. Assoc. Phys. Med*). (A)** Timeframe of each sonochemotherapy treatment schedule, arrows indicate intravenous injection of 0.5 ml SonoVue followed by a 5-ml injection of saline, δt represents the time between each injection; **(B)** Photo of the probe and custom-made probe holder during patient treatment; **(C)** Changes in tumor diameter over time measured from CT images in patients with pancreatic malignancy.

Despite the novelty of the field of ultrasound-mediated drug delivery, a first clinical case study has been conducted in five patients with locally advanced pancreatic cancer (Kotopoulis et al., 2013, 2015). In this study, gemcitabine was administrated by i.v. infusion at a dose of 1000 mg/m^2^ over 30 min (**Figure 7A**). During the last 10 min of chemotherapy, ultrasound imaging was performed in standard abdominal imaging mode to locate the position of the tumor (**Figure 7B**). At the end of gemcitabine infusion, when drug plasma level peaked, 0.5 mL of clinically approved SonoVue^®^ contrast agents followed by 5 mL saline were intravenously injected every 3.5 min to ensure their presence throughout the whole treatment. Tumors were exposed to ultrasound (1.9 MHz, 0.49 MI, 1% DC) using an ultrasound diagnostic scanner. The cumulative ultrasound exposure was only 18.9 s (**Figure 7A**). All five patients tolerated an increased number of treatment cycles compared to gemcitabine treatment without ultrasound (16 ± 7 vs. 9 ± 6 cycles), reflecting an improved physical state as well as an increased survival. In two out of five patients, the maximum tumor diameter was either transiently or permanently reduced, while the other patients exhibited reduced tumor growth compared to a historical control group of 80 patients (**Figure 7C**; Kotopoulis et al., 2013). Compared to this historical data, survival increased with 60% (Kotopoulis et al., 2015). The authors did not report side effects related to this therapeutic protocol. Nevertheless, the true clinical benefit was not clearly established because of the low number of patients studied. The therapeutic protocol (i.e., ultrasound parameters, doses of drug, type and concentrations of microbubbles) should be optimized and long-term safety aspects have to be addressed in future investigations in a larger number of patients.

Moreover, we are referring to a safety study of combining ultrasound microbubbles and chemotherapy to treat liver metastases from gastrointestinal tumors and pancreatic carcinoma conducted by the Profs. K. Yan and L. Shen at Beijing Cancer Hospital (Yan and Shen, 2014). This study is currently recruiting patients. In this clinical trial, gemcitabine will be intravenously injected to patients with pancreatic carcinoma while oxaliplatin and taxol based chemotherapy will be administrated by i.v. perfusion to patients with liver metastases. Thirty min after chemotherapy, 1 mL of SonoVue^®^ contrast agents will be intravenously injected during six times in 20 min. In addition to the safety of the therapeutic protocol, the authors will explore the largest MI and ultrasound treatment time patients can tolerate. The secondary objectives of this clinical trial are to observe the tumor clinical benefit rate and to evaluate the preliminary effects including time to failure and time to death.

## Conclusion

Targeted drug delivery using microbubble-assisted ultrasound has the potential to become a clinically accepted way of improving local anticancer chemotherapy. Although the co-administration approach, using clinically approved microbubbles and free chemotherapeutic drugs, can be seen as the fast-track toward the clinic, the greatest therapeutic potential may lie in the custom-made drug-loaded microbubbles. The latter combines the enhanced vascular permeability and cellular uptake following microbubble-assisted ultrasound with a local release of the drug. However, this implies that new therapeutic particles are to be developed, which require thorough pre-clinical testing for efficacy and safety. A growing number of preclinical experiments have successfully reported the therapeutic benefits of microbubble-assisted ultrasound in the delivery of (anti-cancer) drugs in several animal models. Clinical translation of this method requires further improvements on: (i) the design, characterization, and GMP production of therapeutic microbubbles with prolonged plasma half-life and high drug-loading capacity; (ii) the optimization and standardization of ultrasound parameters used in the field; (iii) the insertion of a medical imaging modality (MRI, ultrasound) to monitor the *in vivo* effects of ultrasound and (iv) the evaluation of drug biodistribution, therapeutic efficacy, and side effects in orthotopic tumor models in small and large animals.

## Conflict of Interest Statement

The authors declare that the research was conducted in the absence of any commercial or financial relationships that could be construed as a potential conflict of interest.
